# Afterhyperpolarization of human motoneurons firing double and triple discharges

**DOI:** 10.3389/fnhum.2014.00373

**Published:** 2014-05-30

**Authors:** Maria Piotrkiewicz, Bożenna Kuraszkiewicz

**Affiliations:** Engineering of Nervous and Muscular System, Nałęcz Institute of Biocybernetics and Biomedical Engineering, Polish Academy of SciencesWarsaw, Poland

**Keywords:** double discharges, doublets, triplets, afterhyperpolarization, delayed depolarization, motoneurons, human

## Introduction

During isometric voluntary contractions of a healthy human muscle, motoneurons (MNs) fire usually with low mean rates, rarely exceeding 25/s (e.g., Garland and Griffin, 1999). However, there are MNs, which sometimes fire double discharges (doublets) with interspike interval (ISI) of few ms. This is observed seldom in normal MNs (e.g., Denslow, 1948; Kudina, 1974; Bawa and Calancie, 1983) but does so more often in neuromuscular disorders (Partanen, 1978; Kostera-Pruszczyk et al., 2002; Piotrkiewicz et al., 2008), where it is considered to be an early sign of MN dysfunction (Rowinska-Marcinska et al., 1999).

Our special attention is paid to the so-called “true” doublets recorded in some MNs under conditions of constant synaptic drive, as opposed to those recorded during rapid contractions (Bawa and Calancie, 1983; Kudina and Andreeva, 2013a). Initial doublets, observed during repetitive movements such as locomotion (Zajac and Young, 1980; Hennig and Lomo, 1987) or respiration (Kirkwood and Munson, 1996), where the synaptic drive undergoes periodic changes, also cannot be classified as “true” doublets. For the sake of clarity, the adjective “true” will be omitted in the further text.

It has been observed that not each human MN is capable of firing doublets (Kudina, 1974; Bawa and Calancie, 1983). By the analogy to animal studies, it was hypothesized that doublets may be generated only in these MNs, which possess the delayed depolarization (DD, Granit et al., 1963; Kernell, 1964; Calvin, 1974) with a prominent hump that may spontaneously cross the firing threshold and evoke an extra spike (Nelson and Burke, 1967; Calvin, 1973). This hypothesis was verified by Kudina and Churikova (1990), who tested the changes in excitability of human motoneurons within ISI by studying the responses of single MNs to the stimulation of Ia afferents. In MNs capable of firing doublets they revealed the period of increased excitability during the first 15 ms after regular discharge, which corresponded well to the duration of DD observed in animal experiments (Granit et al., 1963; Kernell, 1964; Nelson and Burke, 1967; Calvin, 1973).

Nowadays, DD is widely accepted as the mechanism responsible for doublet generation in human MNs (e.g., Kudina, 1974; Bawa and Calancie, 1983; Kudina and Churikova, 1990; Garland and Griffin, 1999). However, it can explain only the origin of occasional doublets, interspersed in regular rhythmic MN activity. Repetitive doublets, i.e., series of doublet-postdoublet ISIs, require some additional mechanism, which would support the DD hump, shown to disappear during MN rhythmic firing (Granit et al., 1963; Calvin and Loeser, 1975). It was recently hypothesized that repetitive doublet firing may be related to plateau potentials (Kudina and Andreeva, 2010, 2013a).

It is still under debate, whether DD is related to other characteristics of a MN, measurable in human experiments. Kernell (1964) noted that DD with a definite hump had a tendency to be more common in MNs with short afterhyperpolarization (AHP), although it could occur with an AHP of any duration. Also in humans, MNs capable of firing doublets were more often found in the fast muscles (Bawa and Calancie, 1983; Kudina and Alexeeva, 1992) than in the slower ones (Andreassen and Rosenfalck, 1980; Kudina and Andreeva, 2013b). For the long time it was believed that the MNs supplying one of the most often investigated slow human muscle, the *soleus*, are devoid of DD. However, it has been recently shown that also MNs supplying human soleus may fire doublets (Piotrkiewicz et al., 2013), which seems to question Kernell's finding. In the present paper we will compare the estimates of AHP duration of MNs capable and not capable of firing double discharges and comment on this controversy. We will also propose a hypothesis on the AHP-related mechanism underlying firing of triplets, which can be observed in some doublet firing MNs.

## AHP duration of MNs capable and not capable of firing double discharges

**Figure 1:**
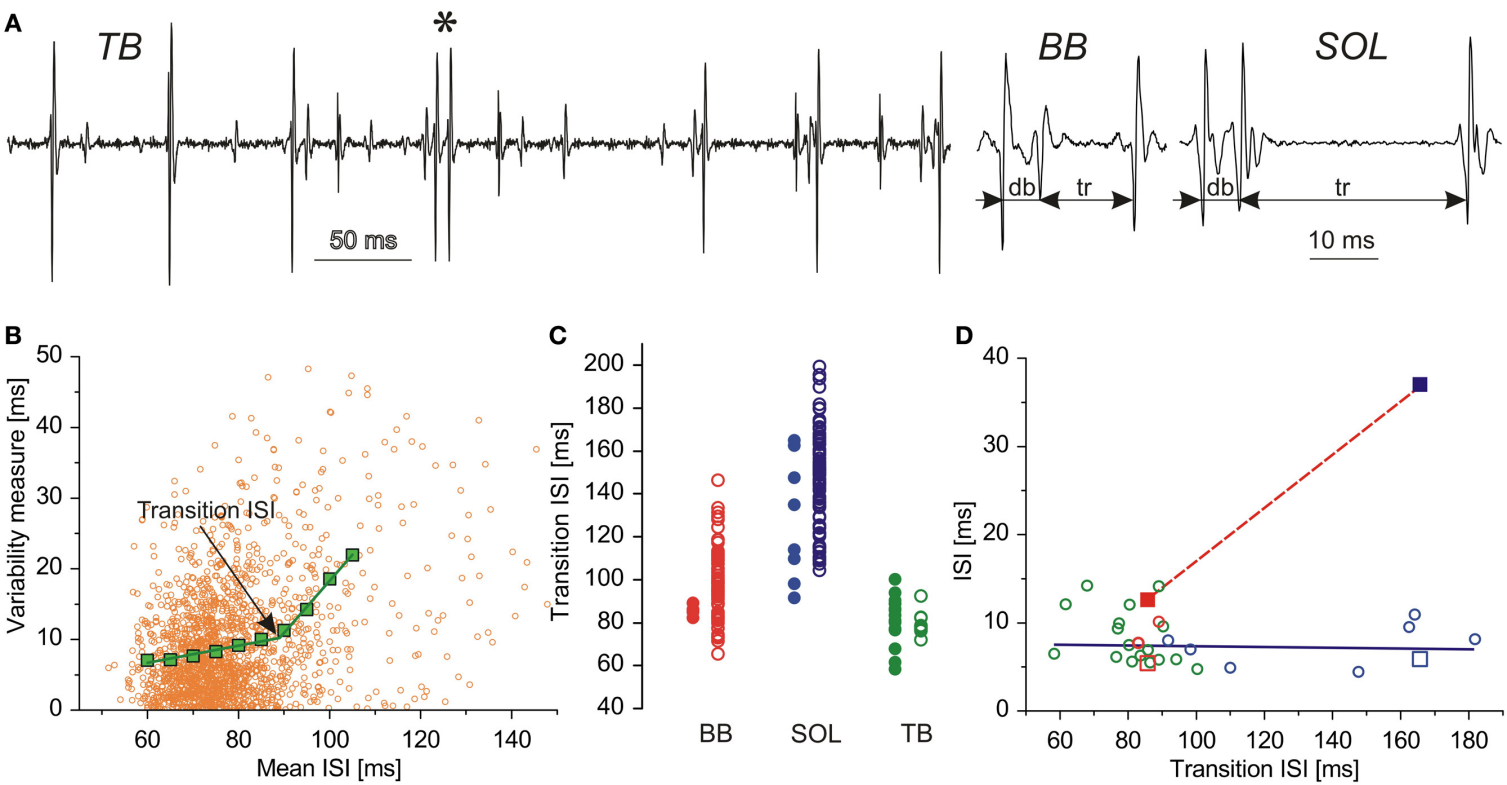
**Double discharges and AHP. (A)** Examples of double and triple discharges, from left to right: single doublet from TB (marked with an asterisk) interspersed in rhythmic MN activity, triplet from BB (11 occurrences), and SOL (6 occurrences); db, doublet ISIs; tr, triplet ISIs; **(B)** Estimation of transition ISI. Ordinate, variability measures: orange dots, *CD*, green squares, *CD_m_*; transition ISI marked by an arrow; abscissa: mean ISI; **(C)** Comparison of transition ISIs for MNs with (heavy circles) and without doublets (open circles); **(D)** Plots of doublet (open symbols) and triplet ISIs (filled symbols) vs. transition ISI: BB, red; TB, green; SOL, blue. Squares, two MNs capable of firing triplets; circles, remaining MNs.

Our opinion is essentially based on the results of the experiments reported earlier (Piotrkiewicz et al., 2001), which were aimed toward investigation of AHP duration in slow (*soleus*, SOL) and fast (*biceps brachii*, BB) human muscle (Exp. 1). Within the motor unit potential trains, recorded in these experiments during steady-state voluntary isometric muscle contractions, we found also doublets and triplets (Figure 1A, Piotrkiewicz et al., 2008). Additionally, we included the results from the *triceps brachii* (TB) from another study, designed specifically for the investigation of double discharges (Kudina and Andreeva, 2010). In this study, the subjects were trained to search for doublet firing motor units. They were instructed to slowly develop a gentle voluntary isometric muscle contraction until a motor unit started firing doublets, and then to keep contraction constant for a few minutes (Exp. 2).

It is commonly accepted that each motor unit discharge is equivalent to the discharge of its MN. Motoneuron AHP duration was estimated from single motor unit potential trains by the method based on the observation of Person and Kudina (1972) that the relationship between the standard deviation and mean value of ISI sharply changes after a certain transition value that was hypothesized to correlate with the AHP duration of the MN. The hypothesis was verified by computer simulations (Piotrkiewicz, 1999) and in the direct recordings from cat MNs (Powers and Binder, 2000), which confirmed this correlation. The AHP duration estimates, which this opinion is based upon, were obtained by the modification of the above method (Piotrkiewicz et al., 2012), using the ISI variability analysis proposed by Holt et al. (1996). The variability was measured as the absolute consecutive difference (CD) between two adjacent ISIs. It was plotted against mean ISI, calculated from the same two intervals (MISI). An example of the plot *CD* vs. MISI is shown in Figure 1B (orange dots). The mean *CD* values (*CD_m_*, green squares) were calculated after grouping the MISIs in 10 ms bins with 5 ms overlap. After rejection of points calculated from insufficient number of *CD* values, the mean data were fit with two linear regression lines and the intersection of these lines used to determine transition ISI, being an estimate of AHP duration.

The AHP duration was estimated for 80 MNs from BB, 90 MNs from SOL, and 28 MNs from TB. From these, MNs firing doublets constituted 5% for BB (4 MNs), 9% for SOL (8 MNs), and 64% for TB (18 MNs). Two MNs recorded in Experiment 1, one from BB and one from SOL, fired also triplets that were much scarcer than doublets: for BB MN 11 triplets vs. 1592 doublets, for SOL MN 6 triplets vs. 342 doublets. Figure 1A (right) presents characteristic triplet firing pattern, which was similar in the two MNs: the ISI between first two components was equal to the doublet ISI, whereas triplet ISI measured between second and third spike was substantially longer (2.4 times for BB and 6.5 times for SOL).

Figure 1C shows the comparison of transition ISIs for MNs with (filled symbols) and without doublets (open symbols) from BB, SOL, and TB. The ranges of both clusters overlap, but the mean values of transition ISIs calculated in each of MN pools of BB and SOL separately are shorter for doublet firing MNs than for MNs without doublets: 85.70 vs. 99.39 and 128.01 vs. 148.04 ms, respectively. Thus, although the AHPs in doublet firing MNs supplying SOL are longer than in their counterparts supplying BB, within the pool of each muscle they tend to be shorter than those in the other MNs.

At the first sight, TB data do not seem to confirm this observation. The difference in mean values of transition ISIs between MNs with and without doublets is negligible: 80.40 vs. 80.00 ms and many of MNs from the former group present transition ISIs longer that those from the latter. However, there are striking differences between TB and BB in doublet incidence and transition ISI ranges. Since both TB and BB are fast muscles, it could be presumed that the AHP duration ranges of their MNs should be comparable. Moreover, it should be noted that the contraction strengths in the Exp. 2 were lower than in the Exp. 1 (below 10 and 30% maximum voluntary contraction, respectively). Thus, the MNs recorded from TB could be expected to have the lower thresholds than those from BB. Consequently, according to the commonly known rules of the orderly recruitment (Milner-Brown et al., 1973a,b; Henneman et al., 1974) as well as to the match observed between motor unit twitch contraction time and the AHP duration of its MN (Kernell et al., 1999), one could expect that AHPs estimated for TB MNs would be rather longer than those of BB MNs. Despite these expectations, the transition ISIs in TB not only are shorter, but their range is much narrower than that of BB MNs.

The differences observed could be related to the differences in experimental protocols, specifically to the different MN sampling. Whereas the sampling in the Exp. 1 could be assumed to be random, in Exp. 2 it was influenced by the training for search of MNs with doublets. It is impossible to say, what happens with MNs during training. It must involve some changes in synaptic inflow, since the majority of doublets recorded in Exp. 2 were repetitive ones. However, it may also be presumed that due to this training the MN sample was limited to those cells, in which finding hump-like DD was most feasible, i.e., to the MNs with shortest AHPs. The narrow range of transition ISIs and the very high proportion of doublet firing MNs in TB sample (64%) are in favor of this presumption. Summing up, the results from all three muscles seem to be in line with the earlier observation of Kernell (1964).

## Interspike intervals of double and triple discharges vs. AHP duration

Figure 1D presents the dependency of the intervals between spike components of doublets and triplets on the AHP duration. The doublet ISI duration of MNs supplying all muscles investigated was independent of transition ISI (open symbols). This result is in line with the suggestions of Granit et al. (1963) and Kernell (1964) that DD may not be the “true” afterpotential. The more recent studies on the ion channels involved in the generation of MN rhythmic firing confirm that the mechanisms responsible for AHP and DD differ from each other (Viana et al., 1993; Kobayashi et al., 1997).

In contrast to the doublet ISI, the triplet ISI measured for SOL (blue filled square) was substantially longer than that measured for BB (red filled square). This observation may suggest that its firing might not be caused by DD, but by the later hump in the time course of AHP conductance described by Baldissera and colleagues (Baldissera and Gustafsson, 1974; Baldissera and Parmiggiani, 1979). This hump has been shown to coincide with the AHP time-to-peak, which would be longer in MNs with longer AHPs. Moreover, AHP summation after short doublet ISI may enhance conductance hump. On the other hand, AHP summation would result in diminished DD (Granit et al., 1963), which also indicates that the firing of the third spike related to DD is hardly possible.

## Concluding remarks

Doublets are rarely recorded during the voluntary muscle activity in healthy human subjects. They are generated by the MNs with hump-like DD, whose characteristics, which could be measured in human experiments, are largely unknown. However, the knowledge of the properties of these MNs is important, since their incidence increases significantly in some neuromuscular diseases, where it is considered to be an early sign of MN dysfunction. We believe that although AHPs of doublet firing MNs supplying SOL are longer than those of BB and TB, in each MN pool the doublet firing human MNs belong to those with shortest AHPs, as do the cat MNs with hump-like DDs. We admit, however, that by now the evidence supporting this belief is rather weak.

Triplets are much more scarce than doublets. Thus, collecting bigger experimental material is virtually impossible. We decided to present this opinion hoping that it may inspire other researchers to look for these rare events in their experimental recordings. We are aware that our data are not sufficient to justify solid conclusions concerning mechanism(s) underlying triplet firing. Nevertheless the specificity of the opinion article encouraged us to formulate the above presented speculative hypothesis about the unusual, intriguing phenomenon of triplet firing.

### Conflict of interest statement

The authors declare that the research was conducted in the absence of any commercial or financial relationships that could be construed as a potential conflict of interest.
